# A generational relational model of nature and mental wellbeing: results of a qualitative analysis

**DOI:** 10.3389/fpsyg.2025.1469507

**Published:** 2025-03-24

**Authors:** Hannah L. I. Bunce, Matthew Owens

**Affiliations:** ^1^Neurology, Department of Neurosciences, Somerset Foundation Trust NHS, Taunton, Somerset, United Kingdom; ^2^Department of Psychology, The Mood Disorders Centre, University of Exeter, Exeter, United Kingdom

**Keywords:** nature-connection, wellbeing, qualitative, relational, Cognitive Analytic Therapy

## Abstract

**Introduction:**

There is a developing evidence base for the benefits of natural environments for health and mental wellbeing. However, given the increasing urbanisation of our planet and subsequent disconnection from our natural world, there is a danger that we may ultimately suffer from a nature ‘generational amnesia’. The facets and mechanisms underpinning these relationships are poorly understood and theoretical frameworks are needed to aid further research. There is a paucity of research into the lived experiences of people with good wellbeing and their nature experiences, which has the potential to elucidate key elements of the nature-wellbeing relationship.

**Methods:**

The current study used a qualitative design to explore themes concerning the lived experiences with nature, of 12 people with self-reported good wellbeing. Semi-structured interviews were carried out and data were analysed using thematic analysis.

**Results:**

Two overarching themes of *human-nature relationship* and *self-regulation* encapsulated the data. Within the first, there were two superordinate themes of *developmental* and *nature interconnectedness*. *Self-regulation* consisted of *managing health* and *flourishing*.

**Discussion:**

A theoretical model is proposed to help better understand these relational themes in a generational context. The model is informed by Cognitive Analytic Therapy and attachment theory and generates testable hypotheses for future research.

## 1 Introduction

Mental health problems are highly prevalent (Cao et al., 2024; Kessler and Bromet, 2013; Moreno-Agostino et al., 2021), increasing (Shorey et al., 2021) and lead to significant impairment for individuals (World Health Organisation, 2017). As a result, recent years have seen a rising demand for mental health services (NHS England, 2016) and notably following the last 2 years of COVID-19 lockdowns (Niedzwiedz et al., 2021; Pierce et al., 2020). Concurrently, resources for treatment of mental health problems are limited, resulting in a growing issue of untreated mental illness (Ebert and Cuijpers, 2018; Kessler et al., 2005). At the same time, we are also witnessing a change in contemporary lifestyle that is becoming increasingly urbanised (van der Wal et al., 2021) with reported stress on the rise (Charles et al., 2021).

A critical turning point has been reached where more people now live in urban areas than rural (van der Wal et al., 2021), with demonstrable evidence of a link between common mental health problems and urbanicity (Vassos et al., 2016; Wiens et al., 2017). Even in contexts where nature is more readily available, culture shifts now see more time spent indoors (Frumkin et al., 2017) and increased time on screens (Trott et al., 2022). Furthermore we are seeing a global trend of reduced nature contact for much of the world population (Bratman et al., 2019). Whilst the health benefits derived from a relationship with nature have existed as a tacit understanding passed down through generations as a cultural wisdom (Redvers et al., 2020), there is a longstanding concern that diminishing experiences of nature over time may ultimately result in an environmental ‘generational amnesia’ (Kahn et al., 2009), thereby reducing the potential to receive positive health and wellbeing benefits from contact and connection with nature.

There is a developing body of empirical evidence supporting a range of health benefits gained from nature (Van Den Bosch and Bird, 2018; Keniger et al., 2013; Lackey et al., 2019; Owens and Bunce, 2022b). For example, access to green spaces in urban areas has been shown to lower distress and increase wellbeing (White et al., 2013). Evidence is also developing for the salutogenic effects of nature on a broad range of health areas such as cognitive function (Berman et al., 2012; Bratman et al., 2015; Ohly et al., 2016), mental health (Barton and Rogerson, 2017; Tillmann et al., 2018), physical health and sleep (Jimenez et al., 2021). Given the need for increasing mental health support and the shortage of service provision, coupled with growing evidence in support of nature-wellbeing benefits (Bratman et al., 2015), nature-based interventions (NBIs) are also being developed as an option for prevention and treatment (Owens and Bunce, 2022a, 2022b; Rosa et al., 2021). While evidence is mounting on the likely efficacy of such approaches, much less is known on potential mechanisms involved (Brymer et al., 2019). There have been endeavours and calls to increase efforts to further elucidate mechanisms involved and develop theoretical frameworks (Cleary et al., 2017). For example, recently a nature-based integrated theoretical framework focussing on the biopsychosocial aspects of resilience has been proposed (White et al., 2023).

Traditional theoretical perspectives seeking to understand the nature-wellbeing connection include Biophilia (Wilson, 1984), Stress Reduction Theory (SRT) (Ulrich, 1981), and Attention Restoration Theory (ART) (Kaplan and Kaplan, 1989). The concept of biophilia is derived from a psychoevolutionary perspective that proposes that humans have an inherent adaptive benefit and innate affinity for the natural world. In support of this evolutionary view, it has been shown in an approach paradigm that people tend, on average, to favour natural, over built-up environments (Schiebel et al., 2022). However, biophilia may only be a partial explanation for the positive effects of nature. Haga et al. (2016) for example, found that a white noise stimulus presented to participants had positive cognitive effects dependent on the framing of the source (‘waterfall’ versus ‘industrial sound’), leaving the possibility that benefits of nature may derive from both genetic and learned sources. The results from a recent genetic study showing that affinity to nature is partially heritable support this notion (Chang et al., 2022).

SRT, also a psychoevolutionary account, proposes that natural environments promote recovery from stress and help lower states of arousal through psychological-physiological pathways, thereby reducing stress and negative affect, leading to an increase in positive affect. A number of empirical studies have supported the central tenet of SRT (Imperatori et al., 2023). For example, *Shinrin Yoku*, a Japanese traditional practice of forest bathing, combines concepts of visiting, observing and *being with* the forest, breathing its air and engaging in mindful awareness practices (Hansen et al., 2017; Kotera et al., 2022; Kotera and Fido, 2021; Li, 2023; Morita et al., 2007). An umbrella review of systematic reviews on Shinrin Yoku and its wellbeing effects demonstrated improvements in quality of life and reductions in perceived as well as physiological markers of stress, e.g., cortisol (Antonelli et al., 2021).

ART asserts that through the process of *soft fascination*, one’s attention is captured by the particular natural environmental features (bottom-up processes) and concurrently facilitates directed attention to be rested and restored (top-down processes). ART suggests three other key features that natural environments support; (1) *Being away*; the sense of getting away from something in order to recuperate, (2) *Extent*; the sense of being in spaciousness, which does not have to be vast in reality and (3) *Compatibility*; a sense of natural affinity, of being able to engage with tasks more readily and easily in the natural world. However, the evidence for ART is mixed, providing only partial support for its explanatory power (Joye and Dewitte, 2018; Ohly et al., 2016). Further extensions to the conventional view on restoration that include a social, relational component have also been put forward (Hartig, 2021).

More recent theoretical models include an application of components of self-determination theory (relatedness and goal orientation) to the link between nature connection and eudaimonic wellbeing (Cleary et al., 2017). Understanding more about nature benefits for wellbeing through a positive psychology frame may assist the development of theory and practice. In clinical psychology, whilst traditional therapeutic approaches have focussed on ill health and negative facets of emotional regulation, positive psychology (Maslow, 1954; Seligman, 1998) primarily addresses positive emotion, engagement and meaning (Seligman and Csikszentmihalyi, 2000). Positive psychology aims to challenge the heavy bias towards negative emotion research and raises a fundamental question about good mental health and wellbeing not just being the absence of ill health but encourages ideas of flourishing (Huppert and So, 2013; Vanderweele, 2017) and, of Maslow’s *Self-actualisation* (Maslow, 1954). Research into positive aspects of wellbeing and good mental health has the potential to elucidate key elements that can translate into novel therapeutic targets. For example, NBIs lend themselves well to a positive psychology approach, as contact with nature has been shown to improve positive emotions (Ballew and Omoto, 2018), more than decreasing negative emotions (McMahan and Estes, 2015; Neill et al., 2019), highlighting positive emotion as a potential key mechanism in improving wellbeing in nature contexts (Cameron et al., 2020). Furthermore, such approaches may also have the potential to increase flourishing, through for example, supporting finding meaning, vitality and emotional stability (Huppert and So, 2013). Recently, the positive psychology literature has raised spirituality in nature as a lacking facet of wellbeing (Ryff, 2021). Searching for meaning and purpose in life can be considered components of the nature-spirituality connection and may also encompass perspective shifts and an acknowledgement of self-transcendence (Løvoll and Sæther, 2022).

Although research has evidenced positive associations between nature and wellbeing and health across a wide range of domains, little is known about the experiences of people with good wellbeing and their interactions with nature. Drawing on positive psychology and investigating elements of nature experiences for people with good wellbeing, may lead to important discoveries for future research and recommendations for clinical and public health, including NBIs (Bragg and Atkins, 2016). Numerous qualitative inquiries have made important insights into the lived experience of people in relation to nature (e.g., Birch et al., 2020; Furness, 2021; Hamby et al., 2022; Irvine et al., 2013), yet few have sought to combine environmental and clinical psychological perspectives and none have additionally included only participants with good to high wellbeing. One qualitative study, however, employed phenomenological methodology and drew on psychoanalytic ideas when interviewing nine people to explore their lived experience of the natural world (Schweitzer et al., 2018). The authors argue for a conceptualisation of nature that includes nature as a primary attachment, nature experienced as a secure base, as ‘twinship’, as containing and as embodied.

Qualitative designs can lend themselves to an inductive approach (Lloyd and Gifford, 2024), by investigating potentially novel factors and research targets without imposing a set of theoretical ideas onto the data. Additionally, given the complexity of potential mechanisms underpinning health benefits of nature on wellbeing, an important avenue of research is the lived experience of individuals with good wellbeing in nature. Exploring the ways in which people with self-reported good wellbeing talk about their experiences in nature may explicate mechanisms and other areas for further research to understand active ingredients in the human-nature-wellbeing relationship (Brymer et al., 2019). To address this gap in knowledge, this study employed a qualitative research design, to enable an in-depth analysis of how mental wellbeing benefits occur from time spent in nature, through an exploration of individuals’ lived experiences. We aimed to explore and understand the lived experience of participants with good wellbeing to answer the question; ‘How does nature support mental wellbeing?’

## 2 Materials and methods

### 2.1 Study design and setting

A qualitative design was used to investigate the impact of nature on wellbeing by exploring the lived experiences of people with average to high wellbeing. This design supports a rich and in-depth understanding of the ways in which people talk about wellbeing and nature and how they may experience potential benefits.

For the purposes of this research, ‘nature’ is defined as “any outdoor area with greenery or other natural features that contrast with the built environment” (Lackey et al., 2019). Mental wellbeing is defined using the World Health Organisation’s (WHO) definition of mental health as “a state of mental well-being that enables people to cope with the stresses of life, realize their abilities, learn well and work well, and contribute to their community” (World Health Organisation, 2022). Time spent in nature includes individuals’ interactions with the natural world, from private gardens, potted plants, public parks and natural wildernesses (Hartig et al., 2011).

### 2.2 Participants

Participants responded to the study advertisement, using an online platform and were a nonclinical group from the local university community. To fulfil the study inclusion criteria, all participants scored above 18 (‘low’ wellbeing), on the Short Warwick-Edinburgh Mental Wellbeing Scale (SWEMWBS; range 1–35; Stewart-Brown et al., 2009). There were no other exclusion criteria, in order to encourage a wide range of participants.

### 2.3 Materials

The Short Warwick-Edinburgh Mental Wellbeing Scale (SWEMWBS; Stewart-Brown et al., 2009) is a seven-item scale measuring mainly psychological and eudaimonic (with few hedonic components), of mental wellbeing. Participants were asked to reflect on the last 2 weeks (e.g., “I’ve been feeling relaxed”) and respond using a five-point Likert scale (1 = “none of the time” to 5 = “all of the time”). Scores range from 1 to 35 and suggested cut-offs for possible mild depression, based on benchmarking studies with the Patient Health Questionnaire-9 (PHQ-9) is 18–20 (Shah et al., 2021). A score of 27.5 or higher is indicative of high wellbeing (Ng Fat et al., 2017).

### 2.4 Procedure

Participants gave informed consent, including for their interview to be recorded, before entering the study. Participants initially completed brief questions on their age, gender and completed the SWEMWBS. Participants scoring above 18 were invited to one-to-one interviews which took place on Zoom video calling platform and lasted approximately 30 min each. Semi-structured interviews were based upon 15 open-ended questions, to encourage and facilitate flexibility in dialogue between the participants and researcher. Questions related to participants’ experiences of time spent in nature and the effects on their mental wellbeing. Interview framework:

1. What does nature mean to you?
2. How often do you spend time in nature?
3. What are the usual locations you visit?
4. What are your main reasons for going to spend time in nature?
5. What are the ways you interact with nature? What activities do you do there?
6. Please describe in detail how you feel when spending time in nature and how you experience natural environments?
7. What is the predominant sense whereby you interact with nature? Touch, hearing, sight
8. How do you tend to feel after you have spent time in nature?
9. To what extent would you say that you experience mental benefits from spending time in nature? How would you describe this?
10. What is it that you think causes this (any) effect in yourself?
11. Would you say these benefits are immediate? Short term? Long lasting? Cumulative?
12. Is mental wellbeing a factor that influences your decision to spend time in nature?
13. Could you describe any differences between how you feel in natural environments vs. built environments?
14. Do you have a sense of personal connection to nature, and could you describe this?
15. What do you think about humans’ relationship with the natural world more generally?

Interview recordings were anonymised and transcribed verbatim by the researcher. Throughout the data collection and analysis process, the researcher kept a reflexive journal (Ortlipp, 2008) to record study reflections to support transparency and objectivity. After the study, participants were fully debriefed and were able to ask any questions in relation to the study. Participants were signposted to wellbeing services as a standard measure. Participants did not receive any remuneration.

### 2.5 Data analysis

Transcripts were coded by one researcher (Bazeley and Jackson, 2019) and key themes were identified and defined using thematic analysis (Braun and Clarke, 2006) to provide a rich description of people’s mental wellbeing experiences after spending time in nature. Qualitative alternatives to the traditional concepts of reliability and validity proposed by Lincoln and Guba (1985), were adhered to. An inductive analysis approach was used to code the data, seeking to describe the dataset as a whole rather than fitting them into predetermined codes (Braun and Clarke, 2006). Nvivo 1.7.1 software was used to collect recurring motifs in the data by looking for word repetitions, metaphors and analogies and examining linguistic features (Ryan and Bernard, 2003). Analysis was guided by a critical realist approach (Bhaskar, 1978; Easton, 2010), which asserts that a single reality exists separately to a multitude of individuals’ experiences that are construed rather than constructed. This enabled the researcher to understand the meaning-making of participants’ experiences in nature.

Data extracts were coded to “capture[s] something important about the data in relation to the research question” (Braun and Clarke, 2006). Braun and Clarke’s (2006) six-phase guide was followed. Phase one involved familiarisation of the data. Repeated readings of the transcripts supported a broad sense of the data and initial notes of interesting patterns and meanings were made. Phase two involved generating initial codes through giving ‘full and equal attention’ to the whole data set, thereby identifying features of the data that were interesting to the analyst. Phase three comprised of organising the full set of codes into themes. Thematic maps and tables were produced to aid analysis at this stage. In phase four, candidate themes were reviewed and refined until the coded data were captured satisfactorily. Phase five ‘defined and refined’ themes to include a narrative with supporting data extracts. Phase six culminated in the production of a report explaining extracts within an analytic narrative, constructing an argument in relation to the research question.

## 3 Results

Twelve people (nine women and three men), between 24 and 53 years of age participated in the study. The data from 12 interviews were analysed as described and resulted in two overarching themes: Human-nature relationship and Self-regulation. These were categorised into four superordinate themes and further subordinate themes. Table 1 shows a breakdown of the themes.

**Table 1:** Levels of themes in the analysis.

Themes				
Overarching	Human-nature relationship		Self-regulation	
Superordinate	Developmental	Nature interconnectedness	Managing Health	Flourishing
Subordinate	Childhood nurturing Childhood nostalgia	Bodily Spiritual Nature opportunities	Prevention Intervention	Solitude Relaxation Stimulation

### 3.1 Human-nature relationship

#### 3.1.1 Developmental

This theme reflects the development of a relationship with nature and one’s attachment to it. Within this theme, participants discussed subthemes of *childhood nurturing* and *childhood nostalgia* in their experiences with nature and wellbeing.

##### 3.1.1.1 Childhood nurturing

*Childhood nurturing* captured the reflections of participants who were parents, and the ways in which they sought to provide nature experiences for their children. Participants cited ‘importance’ as a reason for fostering nature experiences for their children, as well as an acknowledgment that a counter to a perceived increase in gaming and technology-based lifestyles is necessary through accessing nature. Other comments reflected motivations to entertain children, with a tacit understanding of health benefits “it makes me feel better” (Ppt 12) and “I think for their mental health it’s really, really important to get them out, and they always love it when they go.” (Ppt 8). Parents also wanted to provide experiences of freedom and exploration which was found in nature “because we go down on a regular basis, the children always say ‘let’s go to [removed for anonymity]’ so they associate [removed for anonymity] with being free and being able to explore.” (Ppt 4).

##### 3.1.1.2 Childhood nostalgia

*Childhood nostalgia* reflects the ways participants discussed their own childhood experiences with nature. Participants talked about childhood holidays in nature and described fostering a connection with nature, “we used to go down to Cornwall, every year, quite a lot, and my stepdad had a fishing boat, and so we spent hours and hours on the water, so I love the water” (Ppt 10). For participants who grew up in the countryside, talk reflected a sense of returning ‘home’ and associated feelings of peace, “it’s just being home again and, yeah, memories and feelings of peace.” (Ppt 11). For others, there was a realisation that a disconnection had occurred since living away from nature, even though they may have actively sought out and enjoyed a change initially, “I left to go to university in London and I loved it,” (Ppt 10) or became caught up in modern day living, “you’re working away and working in the system” (Ppt 2). Forging a ‘childhood association’ with nature was linked to spending time in nature as a child and recognising feeling more relaxed and comfortable in natural environments “I love the buzz of the city and a town, but I’m definitely more relaxed where there is nature and fewer people.” (Ppt 8). “I didn’t realise how much time I spent in the countryside, when I was younger and then, when you have your 20s and you’re working away and working in the system you then realise how much how disconnected you become from it” (Ppt 2).

#### 3.1.2 Interconnectedness

The human-nature relationship is also defined by connectedness and ways in which participants connect with nature. This superordinate theme is comprised of the bodily experience of nature, the spiritual experience of nature and prioritising nature.

##### 3.1.2.1 Bodily experiences

Participants described connecting with nature through their senses and mainly talked about sight and touch as the principal modalities. “if I think of nature, I have images.” (Ppt 6), “I think it’s probably the sight and the feel in combination. So I really love seeing things grow, particularly this time of year. You get new life, both in flora and fauna, which is really lovely, so I would say visually but also very tactile with the gardening side of things.” (Ppt 8). “when I go out to [removed for anonymity] I see it as an opportunity to reconnect, take my shoes off and ground myself. And I particular[ly] like going in the water, even if it’s freezing cold you get that sort of strong feeling of immersion.” (Ppt 4). Some participants made reference to noticing the sounds of birds “there were just thousands upon thousands of birds. It was a cacophony of noise” (Ppt 10) and of olfactory awareness “the sound of the sea, the sounds of the pebbles moving and so on, but there’s also a smell, which I love.” (Ppt 10).

##### 3.1.2.2 Spiritual experiences

This component of nature experiences was described by participants in contrast to towns, “But there’s no spirituality in a town which you get when you’re out in nature. […] I feel connected with the land around me. It’s almost slightly spiritual.” (Ppt 10). “there’s something in nature that, there is some kind of intelligence or wisdom in nature” (Ppt 4). “there’s a lot of ancient cultures, when I think of nature, I always think of like people like the native Americans who seem to understand this connection and how important it was and they developed a lifestyle, where they could feel close to nature and look after it” (Ppt 4). Some participants talked about perspective and themselves in relation to nature, “I think for me it puts things in perspective, and so I guess […] for it to do that, I must feel that I have a connection somewhere in that process. Nature often makes me feel quite small and sometimes that helps because you realise there’s all this other stuff going on” (Ppt 12). “Especially when I’m by the sea I’m aware that I’m just [a] speck if you see what I mean? I’m here and then I’ll go one day. And it’s quite majestic and important to me.” (Ppt 10). “I think that feeling of it being bigger than you, and it’s always there and its always going on and its being going on since the beginning of time. So then when you’re experiencing it you’re like wow, I’m part of it. You’re part of this thing that’s being going on forever, and you’re just looking at a snapshot of it.” (Ppt 3).

##### 3.1.2.3 Nature opportunities

This is a key theme in participants’ descriptions of their interconnectedness with nature. Participants spoke about a need and importance to prioritise and go into nature regularly, (“integral to my life”; Ppt 1) and this was often facilitated by having dogs and/or children, “And because we’ve got a dog, we go walking in the woods every day, and it’s important for me to be outside at least a bit every day” (Ppt 9), and “Daily. We’ve got a dog, so I walk her a lot,” (Ppt 3), “I’m outside walking in the countryside everyday with my dog, and I take my children to the beach, to the coast to the moors as often as possible” (Ppt 11). Having opportunity for regular contact with nature was valued and vital to participants, “nature is important to me. I have the opportunity to be daily in nature.” (Ppt 7). “I think without that connection we cannot appreciate what it is and what it actually does for us and therefore it’s easy to destroy it or take it for granted.” (Ppt 12).

### 3.2 Self-regulation

#### 3.2.1 Managing health

Within this theme, subthemes of prevention and intervention were identified in participants’ talk of their lived experiences in nature. Whilst it is recognised that there is a continuum between prevention and intervention, themes have been organised to capture participants’ descriptions of intervention and prevention in relation to health.

##### 3.2.1.1 Prevention

Participants acknowledged the role of contact with nature in preventing ill health, “But yeah, definitely, definitely important to me. I think if I didn’t have it, […] if I wasn’t able to go out and walk for half an hour, I think it would have an impact on my well-being and mental health” (Ppt 11). Similarly, participant 7 describes, “It’s difficult to distinguish because I think I’d probably go stir crazy, if I’d only been out once.” Another account focuses on the self-regulatory element of prevention, “I kind of feel like, I am my most full self when I’m connected to nature, and so it’s an innate need, I would say, to emotionally regulate, to physically regulate, because I evolved in a natural environment, like, I have to re-enter that natural environment for effective homeostasis” (Ppt 6).

In preventing ill health, participants also noticed mindfulness qualities within nature experiences that support managing thoughts and feelings, “I was watching a snail yesterday crossing the path, and just little small things like that just gets you out of your four walls and your assignments and it just gives you a change of scenery and gives you a chance to clear your head” (Ppt 11), and, “in a natural environment I’m more inclined to notice little things, small things, small changes, or just the way something looks or the way the light falls” (ppt 12). For others, maintenance of health through nature was cited, “I more just try to do it regularly to maintain that feeling [not stressed and low]” (Ppt 9), and, “it’s like a really big part of how I maintain good mental health, definitely. Even because now my work, I work in mental health as a support worker and some days are really, really heavy, and I’ll often go for a long walk with the dog, or go for a run, and it kind of like shakes it off, you know. Yeah, it’s definitely one of my coping mechanisms that are really important to me.” (Ppt 1). “Having that time each day to go out and walk is a bit of a reset. I think if I hadn’t been doing that, I’d have been mulling things over a lot more, and getting more worried and anxious. So I think its saved me to a certain extent having that as a daily practice” (Ppt 3).

##### 3.2.1.2 Intervention

Participants talked about going into nature “mainly because I know it makes me feel better.” (Ppt 12), and as a response and an attempt to actively manage stress, “I’ve gone for a walk if I’ve had a really stressful day or I find it really, like, relieving” (Ppt 5) or, “it helps me switch off from the preoccupations that I have when I’m at home, so […], if I’m feeling emotionally stressed then it takes me away from those relationships that are stressful. And if I feel stressed about work, then it takes me away from the screen of my laptop, which is kind of my work world,” (Ppt 6). “I think it’s definitely like a sort of de stressor” (Ppt 2).

For one participant, they actively sought nature in response to postnatal depression symptoms, “I remember saying to my husband ‘right, I’m pretty sure I’ve got a bit of postnatal depression, and what I’m going to do this. I’m going to not eat, like, a whole bar of chocolate every day because I don’t think that’s doing me any good. And I’m going to make sure that me and the kids go out for a walk with them in the buggy so I’m actually walking [in nature]” (Ppt 9).

#### 3.2.2 Flourishing

Participants spoke about contact with nature supporting their wellbeing in the *absence* of ‘ill health talk’. This theme has been named ‘flourishing’, as participants described seeking different facets within nature, such as stimulation, relaxation and solitude. In this way, participants were engaging in self-regulation, meeting their needs in pursuit of higher order, flourishing elements of wellbeing.

##### 3.2.2.1 Stimulation

Participants talked about seeking stimulation and being energised by the natural environment, “It’s like if you go camping. If you spend a whole week camping you get like a really big dose of outdoors life. And that lasts, I’m sure that lasts at least a few days after you get home, and kind of energizes you” (Ppt 4), and, “I like outdoor sports, or it goes hand in hand, and I like being in fresh air more than I like being indoors.” (Ppt 7). Participants also talked about associated feelings of connection, contentedness and calm, “I feel energized I also feel kind of deeply connected” (Ppt 6) and, “Before we go out for a walk I always feel a little bit excited and then after we come back, I think the best word I could use is just content. Just feel really content and calm” (Ppt 9).

##### 3.2.2.2 Solitude

Participants talked about seeking solitude in nature and its relationship with being able to think, “I like being on my own […], being able to think and stuff” (Ppt 1). “Just to work through ideas. It’s just a good time” (Ppt 3). Participants also talked about engaging in other activities in nature, to be solitary, “if I go to run outside it’s because I find that running is the only time I don’t think about anything else. But ultimately, it’s to spend time on my own” (Ppt 5).

##### 3.2.2.3 Relaxation

Participants described feelings of relaxation whilst in nature, in the absence of ill health, “I think it’s really relaxing. I think it makes you put things in perspective and it’s very grounding” (Ppt 8). “To me that’s like […] a mini version of nature where we can like just relax by just digging and planting and watching things grow and looking at bees and just having that kind of connection with things that kind of get you out of your head and back into your body” (Ppt 4). Some participants discussed feeling relaxed in conjunction with rejuvenation, in nature, “I find it really rejuvenating. It’s beautiful, so I think those two things go hand in hand, and when I see the beauty of nature, I feel refreshed and energized, more relaxed” (Ppt 6).

## 4 Discussion

This study explored the lived experiences of participants with good wellbeing in nature. The aims were to elucidate the ways in which participants discussed their nature experiences in the context of their wellbeing to further understanding of eudaimonic nature benefits. Nature experiences from the data corpus were broadly grouped into two overarching themes: the human-nature relationship and self-regulation (Table 1).

### 4.1 The human-nature relationship

In considering the human-nature relationship, two key themes are (1) development of nature relationships and (2) factors important to nature interconnectedness.

Taking a developmental perspective, themes from the data encapsulate the notion that the human-nature relationship may be fostered in early years, throughout childhood similarly to that of a caregiver and child as described by attachment theory (AT; Ainsworth et al., 1978; Bowlby, 1969).

#### 4.1.1 Childhood nostalgia

Characterised as a longing for the past, nostalgia is often described as a bitter-sweet emotion (Sedikides and Wildschut, 2018). In moderate quantities, it can represent a positive emotion, with beneficial effects on psychological wellbeing. However, the propensity for nostalgia may become a negative experience, akin to depressive rumination, adversely affecting wellbeing (Newman et al., 2020; Routledge et al., 2013). For most participants in the present study, however, the recollection of the presence of nature in a reliable and predictable way, in their formative years presented a secure base to which they could return (Ainsworth et al., 1978; Jordan, 2009). This is consistent with recent qualitative work on childhood experiences that found that participants primarily used positive phrases to describe childhood interactions with nature with many saying they were a “highly influential” aspect of their upbringing (Hamby et al., 2022).

For some, there was an acknowledgment of initially wanting to explore urban spaces in emerging adulthood years and then a return ‘home’ to nature and the countryside in later years. Having developed a secure nature-base appeared to allow participants to (a) provide themselves with regular nature opportunities as adults (Stehl et al., 2024; Vitale et al., 2022) and (b) access nature to self-regulate. Indeed there is evidence that engaging in nostalgia, can effect change on the future through reflecting on the past (Boym, 2001). Nostalgia towards specific environments is also closely related to the theory of place attachment. This suggests that pleasurable experiences with certain places as a child leads to strong, long-term, and even emotional bonds to such places (Morgan, 2010). Furthermore, it has been suggested that nostalgia may mediate the link between the environment and the restorative effects it can bestow (Yan et al., 2023).

#### 4.1.2 Childhood nurturing

Parents that were provided opportunities for the development of their own secure nature-base as children endeavoured to foster and nurture a nature connection for their own children. They cited reasons of importance and described the change in societal context of hyper-technology opportunities and demands (for example, gaming and online communication). This may be conceptualised as ‘nostalgia-in-action’, as parents seek to provide a secure nature-base and counter the perceived ‘ills’ and ‘technostress’ (Anderson, 1985) of modern-day living, thereby contributing to the development of a nature relationship. Furthermore, parents may be tapping into a biophilic (Wilson, 1984) ‘knowing’ of the importance of the nonhuman world on an infant’s (and beyond) healthy development (Searles, 1960). This and other themes described in the present analysis are consistent with previous phenomenological analyses drawing on psychoanalytic concepts (Schweitzer et al., 2018).

#### 4.1.3 Bodily experiences

In line with previous studies (Beery and Jørgensen, 2018), nature was experienced through the ‘sensing body’ (Merleau-Ponty, 1968) and particularly the visual and tactile systems, from which we make sense of the world around us. This is also consistent with research suggesting a significant bias towards the visual systems (Franco et al., 2017). Participants wanted to engage with the physical elements of nature, for example, by taking their shoes off to connect with the earth or immersion in cold water. This ‘*re-connection*’ as it was described, suggests a very concrete method to engage with nature, a *returning to* nature, which has been reported in previous qualitative studies (Furness, 2021). Descriptions of experiencing nature visually evoked mindfulness qualities or that of forest bathing (Clarke et al., 2021), as participants noticed the detail of flora and fauna around them and were able to hold present moment awareness.

#### 4.1.4 Spiritual experiences

Similarly to previous bodies of work (e.g., Irvine et al., 2013; Naor and Mayseless, 2020), participants spoke about ‘*being* with nature’ that was more than a cultivated present moment awareness, as compared to bodily experiences with nature, and of a self-transcendence. Participants acknowledged an inherent, primeval bond they were connecting to and *being with*, in a distinctly non-physical sense. Spiritual experiences in nature, (compared with other spiritual experiences) are cited as the most common, leading to peak and transcendent experiences that foster a sense of profound connection and oneness (MacDonald, 2009; Naor and Mayseless, 2020). This spiritual facet of the experience may comprise part of the biophilia hypothesis of nature (Wilson, 1984), speaking to humans’ natural affinity. Furthermore, according to Maslow, spirituality is central to our humanness and is key to his humanistic approach (Maslow, 1969). Despite this, spirituality remains a lesser, if not overlooked factor in the wellbeing literature (Ryff, 2021). Participants were aware of their spiritual connection with nature, which may be an important maintaining part of the human-nature relationship. One participant spoke of a ‘wisdom’ in nature and of “communing with that higher wisdom” (Ppt 4).

#### 4.1.5 Nature opportunities

Participants talked about prioritising and valuing nature in their lives, experiencing nature daily for all except two participants, who went into nature most days. Participants in the main either had dogs and/or children, which they often spoke about in conjunction with daily nature visits. The importance of nature contact was stressed by participants, even in the context of busy lives. This was valued as important and necessary to what can be best described as nature interconnectedness. This has been described in previous qualitative work on motivation to access green space (Irvine et al., 2013). Together, experiencing nature physically through the senses (with present moment awareness) and spiritually (having insight into the spiritual connection), in combination with providing oneself with regular (daily) nature opportunities, formed the experience of nature interconnectedness.

Reflecting on an individual’s experience with nature necessitates a relational approach in considering how one *relates to* nature and how this is developed. A relational understanding of humans and nature is not new and is thought to have characterised the salubrious interaction styles of palaeolithic humans (Thomas, 2006), with associated benefits for cohesive living and ‘oneness’ with all living things, including humans and the environment. Ancient environments would have been inherently wilder than our modern-day standards and this decline in ‘wildness’, perhaps combined with the increase in time spent indoors and on screens may contribute to a change in the ways in which we have learnt to relate to nature. As our environments become increasingly urbanised (van der Wal et al., 2021), the increase in our ill health as a species, may be reflected in our inability to access natural environments to reduce our stress, in alignment with SRT (Ulrich, 1981). The qualities of relationships with nature may also be differentiated by culture, with a more interconnected, reciprocal relationship seen in indigenous cultures (Jordan, 2009) and more of a separation from nature in the western world (Schroeder, 2007).

### 4.2 Self-regulation

The salience of the self-regulation theme is consistent with previous qualitative research (Djernis et al., 2023) and theories of nature and self-regulation have been explored in the literature, including nature’s role in affect regulation (Gilbert et al., 2008; Richardson et al., 2016; Ríos-Rodríguez et al., 2024). The superordinate and subordinate themes under ‘self-regulation’ broadly divide into (1) ‘managing health’, through preventative and interventive strategies and (2) ‘flourishing’, including seeking solitude, stimulation and relaxation.

#### 4.2.1 Managing health

Participants actively sought to return to their secure nature-base. This was accompanied by an inherent understanding that their health is better when they do access nature (preventative) or else, nature was harnessed symptomatically to mitigate feelings of stress or low mood (interventive). This notion of turning to nature as a health resource is consistent with previous research (Irvine et al., 2013; Reeves et al., 2021). This can be explained by SRT, as the context of nature may help directly to reduce stress. In addition, ART may explain how participants seek to manage their health in an indirect way, as attention is restored, thus replenishing cognitive reserves and alleviating fatigue on the system, which is known to affect mood (Giallo et al., 2016; Ormstad and Eilertsen, 2015). Interestingly, there is evidence to suggest that visits to green spaces may provide economic benefits through reduced healthcare costs and increased work productivity (Buckley and Chauvenet, 2022).

#### 4.2.2 Flourishing

This superordinate theme encapsulated subordinate categories of seeking *solitude*, *relaxation* and *stimulation*. These are considered as distinct from the ‘managing health’ superordinate theme as they were described in the absence of ill-health talk. Participants sought nature experiences to meet their arousal levels, either wanting relaxation, stimulation and/or solitude. By seeking these states, participants were enabling ‘flourishing’, to attain higher order needs, such as living with vitality, seeking positive emotion and finding meaning. For example, using solitude to reflect and find meaning, or wanting to experience relaxation to find positive emotion (Huppert and So, 2013). Specific routes to flourishing will be individualised (Ashfield et al., 2012) and context-dependent, but this overall pursuit is in line with Maslow’s Self-actualisation, “What humans can be, they must be. The more satisfied, the healthier we are” (Maslow, 1954), and therefore can constitute a form of self-regulation. It has been discussed previously that ART and SRT may not adequately explain the benefits of nature in the absence of ill-health (Johnsen and Rydstedt, 2013), such as seeking stimulation or even relaxation in the absence of stress, and therefore concepts of flourishing and self-actualising may be better placed. Consistent with others we recognise that seeking meaning in life has a central place in wellbeing and includes spiritual experiences of transcendence and the sublime (Løvoll and Sæther, 2022). We also note here that spiritual meaning-making may comprise connection to nature, oneself and others and may or may not be religious in nature (Jirásek, 2023).

### 4.3 Theoretical considerations

The evidence to date highlights the complexity and likely multiplicity of processes in forming a unifying theory of nature benefits on wellbeing (Brymer et al., 2020). However, we believe that the development of relational experience with nature in formative years is fundamental. In our view, the development of a secure nature-base may be best understood through the lens of Cognitive Analytic Therapy (CAT; Ryle, 1995). CAT is an evidence-based and time-limited, collaborative psychotherapy that draws from cognitive, psychoanalytic and developmental theories (Ryle and Kerr, 2020). CAT seeks to address mental health difficulties that are hypothesised to develop from survival coping strategies that have their origins in early childhood relationships and asserts that a child learns how to relate to themselves and others through these early relationships, or ‘reciprocal roles’. Whilst CAT goes beyond AT in synthesising learnt and transmitted cultural meaning and values, it shares some overlap with AT, which is concerned with how a child learns to manage their safety and security in the world, through their early experiences with caregivers. These early experiences create ‘internal working models’ (AT), or a more ‘intersubjective sense of self’ (CAT), which the child reverts to, especially when their unmanageable feelings are activated and is dependent on their relationship to others.

### 4.4 Understanding secure nature-base through CAT

If a child is provided with nature opportunities that support a safe and positive exploration of the natural environment (Bratman et al., 2021); *providing nature opportunities*, the child understands and develops a felt sense of having a nature relationship, through being given opportunities to engage with nature; *being given nature opportunities*. This becomes internalised and thus the child learns how to relate in this way to themselves, others and their environment; that is, they can provide themselves (and others) with nature opportunities. The child can learn the associated feelings elicited when in nature. In this way, caregivers can model to their children how to relate to their natural environment (Windhorst and Williams, 2015; Wu et al., 2023). Indeed, parental connection with nature is a predictor of early childhood nature connection (Barrable and Booth, 2020). In this study, parents provided childhood nature opportunities for their children to scaffold self-regulation. This was often in relation to understanding that a counter was needed against the perceived ills of technology or an inherent, biophilic understanding that it was ‘just good for them’. In this way, parents were providing a *nature-supporting -> nature-supported* reciprocal role for children to experience and internalise (Hinds and Sparks, 2008), which may support future nature contact and connection as adults (Asah et al., 2018; Pensini et al., 2016; van Heel et al., 2023).

Miller (2016) suggests that children are born with an inherent capacity to interact with the transcendent, which may link to the spiritual experiences in nature. It is important to note that children may be empowered or disempowered in their relationships with nature, through the types of modelling caregivers provide whilst in nature. For example, the importance of play has long been understood to support the development of children (Dodd and Lester, 2021; Winnicott, 1971) and play in nature can proffer benefits to child development (Dankiw et al., 2020). Furthermore, there is evidence for children engaging in more relational interactions in ‘wilder’ nature versus more domestic settings (Weiss et al., 2023). Whilst there appears to be much gained from play and nature opportunities for children, it may also be that negative experiences can set up potential biophobic tendencies (Soga and Gaston, 2022) as well as the ‘extinction of experiences’ in nature (Soga et al., 2020). By combining an individual approach examining factors necessary to nature interconnectedness, and a relational, systemic approach considering transgenerational patterns (development of a nature relationship), this can support access to an empowered position of self-regulation.

**Figure 1:**
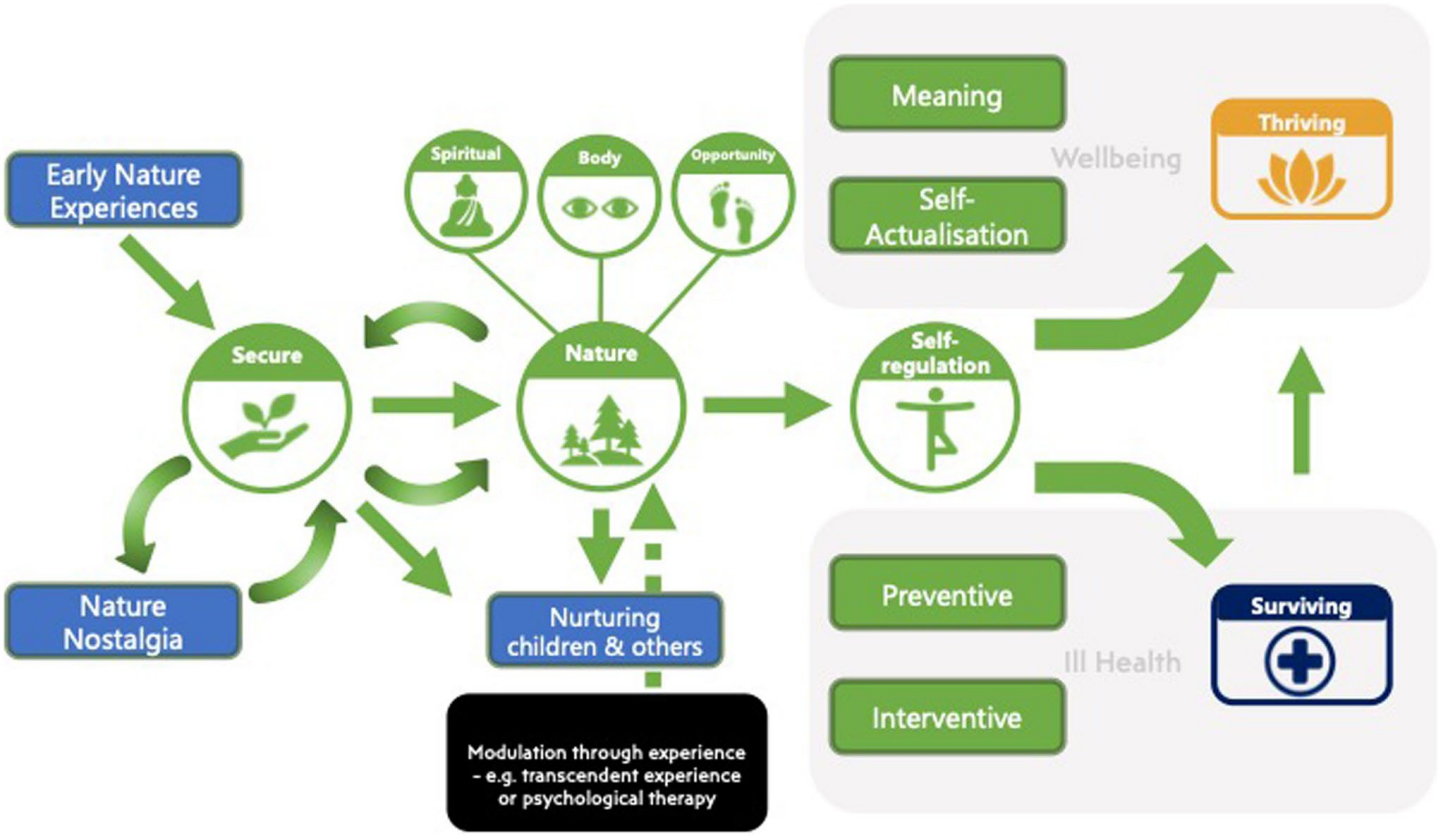
A generational relational model of nature and mental wellbeing.

The present qualitative study has generated a number of themes that have produced testable hypotheses for future research, including quantitative studies. To aid this endeavour we summarise these in a model below that can be used to guide future research to (see Figure 1). Following Schweitzer et al. (2018) we propose that *Early Nature Experiences* can foster the development of a Secure nature-base attachment (denoted as *Secure*). This in turn may lead to Nature Interconnectedness (denoted as *Nature*), which may support *Self-regulation*, which we believe along with others (Djernis et al., 2023; White et al., 2023) is central to the benefits of nature on mental wellbeing. Self-regulation may occur in the context of ill health (via *Preventive* and *Interventive* strategies) or wellbeing (via *Self-actualisation* and *Meaning*). For example, to mitigate psychological ill health an individual may proactively access natural spaces to prevent the unfolding of mental health problems or may seek the therapeutic benefits of nature to self-manage and restore good psychological health, for example when experiencing an existing psychological problem. In this way, individuals can be said to be making an attempt at *Surviving*. Recognising that in the context of average wellbeing there may be a potential to flourish through meaning-making, seeking positive emotion and living with vitality. Here the proposed pathway can be thought of as *thriving*.

Nature Interconnection is supported by prioritising *Nature Opportunities* and engaging in *Bodily* and *Spiritual* experiences (e.g., sublime experiences). Nature interconnectedness and a Secure-nature base can foster the ability to Nurture Nature Experiences and opportunities for children (transgenerational) and for others. In addition, Nature Interconnectedness may be fostered through other processes such as a transcendent experience (multi-factorial) or formal intervention such as psychological therapy. *Nature Nostalgia* may maintain the Secure-nature base and can refer to any early nature experiences, even if experienced later in life.

We recognise that early secure nature attachments may not be formed in all individuals and hypothesise that Nature Interconnectedness may still develop through other environmental experiences across the life span. These may be acquired for example, through posttraumatic growth, sublime experiences or more formal intervention such as public health psychoeducation or psychological therapy. Nevertheless, a clear hypothesis stemming from the model is that those who have developed a secure Nature-base, have high levels of Nature Interconnectedness, will have the highest wellbeing, and will be most likely to show evidence of thriving.

We do not suggest that the proposed model is a comprehensive set of factors involved in the nature-wellbeing connection and believe that other environmental, genetic, social, physiological and behavioural components are also important. However, we believe this to be a useful framework from which to develop hypotheses for empirical testing of the developmental and generational trajectories of Nature Interconnectedness and wellbeing and to ascertain ‘what works for whom’ (Fonagy, 2010) in terms of intervention.

### 4.5 Strengths and limitations

Through a detailed qualitative examination of the lived experiences of nature, a working testable model has been produced to guide future research. This study benefitted from an in-depth exploration of people with good wellbeing and their nature experiences, in alignment with positive psychology, providing an understanding of important factors related to good wellbeing and flourishing. Limitations of the study include the lack of generalisability to other groups, for example, other demographics such as younger people or older adults and non-UK residents. It will be important to explore these questions with other populations, to ascertain whether the findings are replicated. The study findings also highlight individual factors (nature interconnectedness) and systemic factors (development of a nature relationship) that are important to research further, for consideration in interventions.

### 4.6 Clinical implications

Our understanding of the ways in which we can engage with nature and its benefits for wellbeing are developing. Exploring the ways in which our nature relationships develop, using a therapeutic framework may give clinical utility in working, intra-psychologically, inter-psychologically and systemically with individuals and their wider contexts.

Family workers and children and young people’s services could consider assessing for contact with nature within family systems and offer simple suggestions for increasing nature connection and contact, which could be physical, or virtual if inaccessible. This may also be particularly important for people with Adverse Childhood Experiences (Touloumakos and Barrable, 2020). Suitable methods to increase contact and connection could include (but are not limited to) gardening, (which is scalable and affordable, for example planting seeds indoors), attending nature walks or engaging in nature art. Increasing contact and connection with nature may also support individuals’ and families’ prioritisation and valuing of nature, thus increasing nature opportunities in the long-term for self-regulation. Focussing on relational processes, can also lend itself to prevention applications. For example, it may be helpful to develop an intervention in family systems where parents themselves were not afforded nature opportunities when young and so may be unable to provide this for their children, thus potentially maintaining the cycle trans-generationally.

The benefits of nature supporting self-regulation (managing health and flourishing) may also be shared through brief psychoeducation, which can be promoted by healthcare workers and may support pre-contemplation and contemplation stages of change. Social prescribing, whilst still developing an evidence-base (Pescheny et al., 2020) could also be used to encourage adults and children to harness nature benefits, for whom nature opportunities are minimal. When delivering nature-based interventions or traditional interventions in nature, healthcare professionals could support people to consider the bodily and spiritual elements of their nature experiences, to help foster their nature interconnectedness.

### 4.7 Future directions

Future qualitative investigation should explore the experiences and nature perceptions of adults who did not have a secure nature-base nor nature opportunities from which to return to, and the experiences of those that live in the countryside compared with those that live in urban areas. As nature-disconnection is an under-researched area (Barrable and Booth, 2022), these studies could support better understanding of contributory factors and barriers to nature connection. It will be important to test the suggested associations between the themes identified in this study to and to test for the direction of effect. First, testing in quantitative design studies whether a secure nature-base in childhood prospectively predicts whether parents establish secure nature-bases for their children is required. Second, testing whether those with secure nature-bases are better able to self-regulate, within the domains of intervention, prevention, and flourishing is a clear next step. Future NBIs should focus on testing increasing the bodily and spiritual components of nature experiences, as well as increasing prioritisation of nature opportunities. Longitudinal cohort studies could test the transgenerational effects of fostering a secure-nature base proposed in this study.

### 4.8 Conclusion

The development of a human-nature relationship and specifically, a secure nature-base, may be nurtured through childhood nature opportunities provided for by caregivers. These opportunities support nature interconnectedness through bodily and spiritual experiences, facilitated and scaffolded by caregivers. The importance of having a secure nature-base is the ability to support self-regulation (to survive and thrive), using nature whilst growing up and as an adult. These relational patterns can then be passed onto future generations.

## Data Availability

The datasets presented in this article are not readily available because the qualitative raw data generated and/or analysed during the current study are not publicly available due to risk of individual privacy breaches. Requests to access the datasets should be directed to hannah@rowantree.uk.
